# Fine mapping of fatness QTL on porcine chromosome X and analyses of three positional candidate genes

**DOI:** 10.1186/1471-2156-14-46

**Published:** 2013-06-01

**Authors:** Junwu Ma, Hélène Gilbert, Nathalie Iannuccelli, Yanyu Duan, Beili Guo, Weibing Huang, Huanban Ma, Juliette Riquet, Jean-Pierre Bidanel, Lusheng Huang, Denis Milan

**Affiliations:** 1 INRA, UMR444 Laboratoire de Génétique Cellulaire, Castanet-Tolosan F-31326, France; 2 Key Laboratory for Animal Biotechnology of Jiangxi Province and the Ministry of Agriculture of China, Jiangxi Agricultural University, Nanchang 330045, P.R. China; 3 INRA, UMR1313 GABI, Jouy-en-Josas F-78352, France; 4 AgroParisTech, UMR1313 GABI, Paris 05 F-75231, France

**Keywords:** Pig, QTL, Fatness, X chromosome, Coldspot, Meishan, Erhualian

## Abstract

**Background:**

Porcine chromosome X harbors four QTL strongly affecting backfat thickness (BFT), ham weight (HW), intramuscular fat content (IMF) and loin eye area (LEA). The confidence intervals (CI) of these QTL overlap and span more than 30 cM, or approximately 80 Mb. This study therefore attempts to fine map these QTL by joint analysis of two large-scale F_2_ populations (Large White × Meishan and White Duroc × Erhualian constructed by INRA and JXAU respectively) and furthermore, to determine whether these QTL are caused by mutations in three positional candidate genes (*ACSL4*, *SERPINA7* and *IRS4*) involved in lipid biosynthesis.

**Results:**

A female-specific linkage map with an average distance of 2 cM between markers in the initial QTL interval (*SW2456*-*SW1943*) was created and used here. The CI of QTL for BFT, HW and LEA were narrowed down to 6–7 cM, resulting from the joint analysis. For IMF, two linked QTL were revealed in the INRA population but not in the JXAU population, causing a wider CI (13 cM) for IMF QTL. Linkage analyses using two subsets of INRA F_1_ dam families demonstrate that the BFT and HW QTL were segregating in the Meishan pigs. Moreover, haplotype comparisons between these dams suggest that within the refined QTL region, the recombination coldspot (~34 Mb) flanked by markers *MCSE3F14* and *UMNP1218* is unlikely to contain QTL genes. Two SNPs in the *ACSL4* gene were identified and showed significant association with BFT and HW, but they and the known polymorphisms in the other two genes are unlikely to be causal mutations.

**Conclusion:**

The candidate QTL regions have been greatly reduced and the QTL are most likely located downstream of the recombination coldspot. The segregation of SSCX QTL for BFT and HW within Meishan breed provides an opportunity for us to make effective use of Meishan chromosome X in crossbreeding. Further studies should attempt to identify the impact of additional DNA sequence (e.g. CNV) and expression variation in the three genes or their surrounding genes on these traits.

## Background

Quantitative trait loci (QTL) for fat deposition and muscle masses have been consistently identified proximal to the centromere of porcine chromosome X (SSCX) in a variety of crosses between Western breeds and Chinese Meishan (MS) [1-8]. The most significant result was obtained by Milan *et al.*[4], who reported that the backfat QTL accounted for almost 40% of the phenotypic variance in a Large White (LW) × MS F_2_ population which was set up at INRA in France. However, another study showed that the SSCX QTL for backfat thickness (BFT) seemed to be absent in another LW × MS F_2_ population developed at the Roslin Institute in UK [9], suggesting that QTL alleles might segregate within LW or MS, or both breeds. Recently, a large-scale F_2_ population (>1000 F_2_) was produced by crossing White Duroc (WD) to Chinese Erhualian (ER) at Jiangxi Agricultural University (JXAU) in China. ER and MS pigs are two sublines of the Taihu breed known for high fertility, and WD is the result of crossbreeding Duroc with LW or Landrace breeds. A genome-wide QTL analysis using this F_2_ population also identified QTL for fatness and muscling traits on SSCX [10].

Joint analysis of two or more genetically similar populations can potentially lead to more precise estimations of the location and effect of a common QTL, or to identification of differences in the QTL effects among different populations [11-13]. A previous joint analysis of 5 different crosses involving six breeds (wild boar, LW, Landrace, Iberian, Pietrain and MS) suggested that the SSCX region between *SW2456* and *SW1943* (~40 cM on female-specific linkage map) contains one highly significant QTL for BFT and a distinct QTL for ham weight [14]. Furthermore, the fatness allele at the BFT QTL was regarded to be of Asian origin, while the ham weight QTL seemed to be segregating only in crosses involving European breeds. However, the precision of the QTL location estimated by this joint analysis was still unsatisfying, due to low-density linkage map, small number of common markers and differences in trait measurements applied in different crosses.

Long-chain acyl-CoA synthetase family member 4 (*ACSL4*) is a strong functional candidate for the fatness QTL on SSCX. *ACSL4* plays a key role in the metabolism of fatty acids and thus in the energy balance of the organism. The porcine *ACSL4* gene is located very close to the most likely position of the QTL affecting fatness and muscling traits in a Wild Boar × Meishan F_2_ family [2]. Furthermore, polymorphisms of the *ACSL4* gene were reported to be associated with the percentages of oleic fatty acid and monounsaturated fatty acids in an Iberian × Landrace F_2_ population, in which QTL for the two traits had been detected on SSCX [15]. Nevertheless, no significant QTL effect on BFT was found in this population. So far, the effect of the *ACSL4* gene on BFT has not been clarified in any QTL mapping population.

Besides *ACSL4*, the insulin receptor substrate 4 (*IRS4*) and serpin peptidase inhibitor, clade A (*SERPIN7*, also named as *TBG*) genes were also proposed as positional and functional candidate genes for the SSCX QTL [2]; and polymorphisms in them were found to be significantly associated with BFT measured in MS × Western breed pedigrees [16-18].

In this study, we carried out single and joint analyses of the aforementioned INRA and JXAU populations to refine the SSCX QTL for fatness and muscling traits, using a linkage map with high-density markers that were located within the initial QTL confidence interval (*SW2456*-*SW1943*), and genotyped in the two populations. Further refinements are suggested after an analysis of the haplotypes segregating in the INRA F_1_ sows. Moreover, we examined the possible implication of the *ACSL4*, *IRS4* and *SEPINA7* genes’ polymorphisms on BFT and ham weight (HW).

## Results

### Marker selection and genotyping

Previously, only five to seven markers on the SSCX were used for the genome-wide QTL mapping in each population. In order to increase the marker density, 46 markers including microsatellites and SNPs were chosen from the USDA-MARC map (http://www.thearkdb.org/arkdb/do/getChromosomeDetails?accession=ARKSPC00000001), from the literatures [16,17,19-23], or developed within this project [24]. The Additional file 1: Table S1 shows the information about the locations and derivation of these markers. Their polymorphisms were assessed using both INRA and JXAU F_0_ and F_1_ animals. Based on the polymorphisms and the physical distribution of all markers, 19 markers were selected to be further genotyped in all F_2_ animals. Finally, a total of 22 markers were common to both populations, namely: 19 microsatellites (*SW980*, *SW1903*, *SW2456*, *SWR1861*, *SW259*, *SW1994*, *SW1426*, *SW1522*, *SW1943*, *SW1608*, *UMNP1174*, *UMNP1218*, *UMNP71*, *UMNP891*, *UMNP93*, *MCSE231M24*, *MCSE347J6*, *MCSI0244D12*, *MCST96O22*), 2 SNPs in the *ACSL4* gene (intron3:g.280G > A and g.359A > C, denoted as *ACSL4I3B280R* and *ACSL4I3B359M* , respectively; the allele “1” of the two SNPs represents the nucleotide “G” for *ACSL4I3B280R* or “A” for *ACSL4I3B359M*; GenBank No. AJ785784) and a 14-bp deletion mutation in the *SLC25A5* gene (intron2:g.103-116del 14, denoted as *SLC25A5I2B102DE*; the allele “1” represents the non-deleted allele, GenBank No. AM746979). The 22 markers are all located in the chromosome X-specific region, i.e. non-pseudoautosomal region.

### QTL analyses in populations

**Figure 1:**
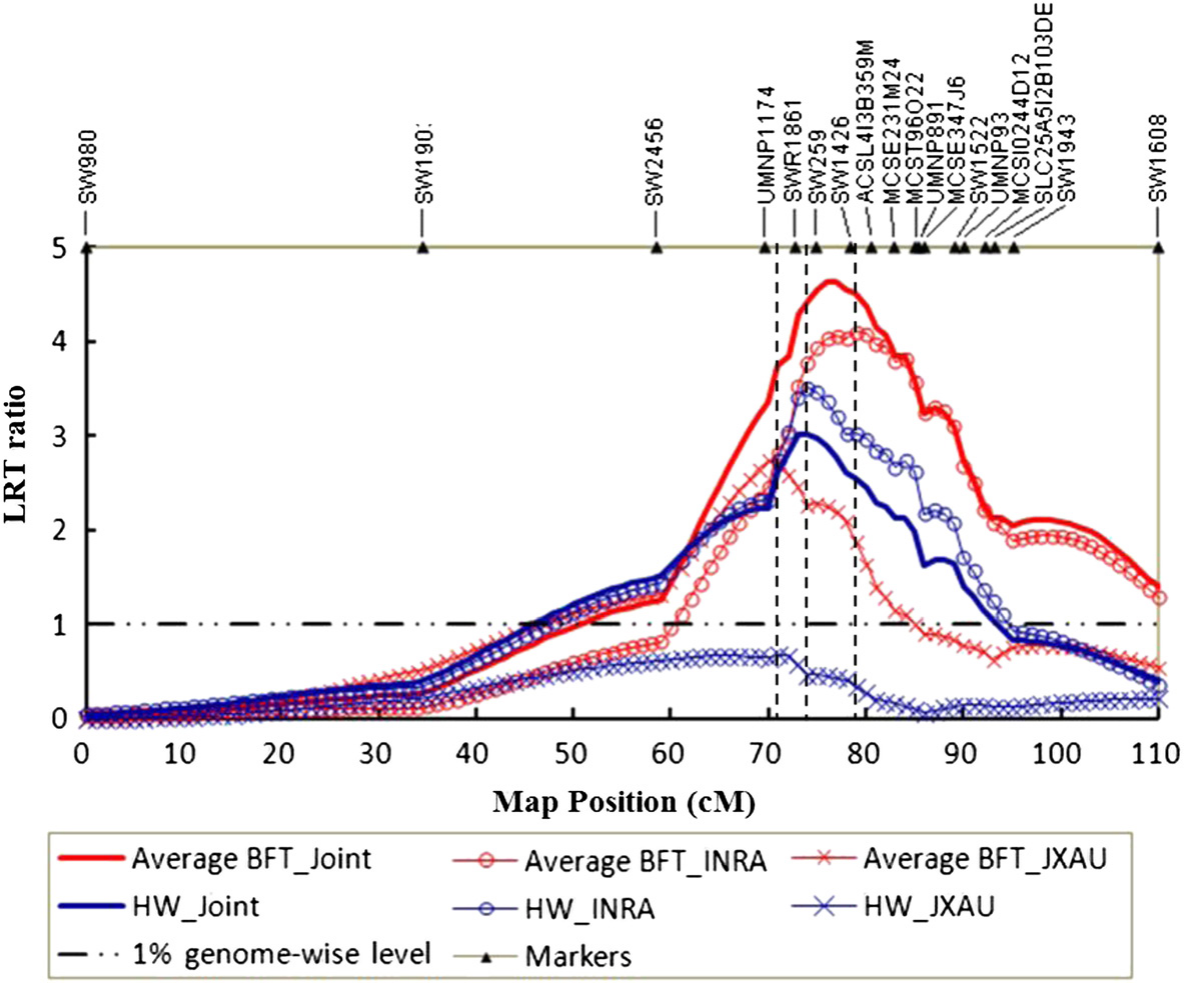
**Profiles of the test statistics across the porcine chromosome X for average backfat thickness (average BFT) and ham weight (HW) in individual and joint populations with line-cross model.** Maximum LRT locations for BFT and HW of JXAU (71 cM), for HW of INRA (74 cM) and for BFT of INRA (79 cM) are indicated by three dash lines. The “LRT ratio” (y-axis) represents a ratio between LRT of QTL and the 1% genome-wise significance threshold obtained for the QTL detection in individual or joint populations.

The results from the single-population analyses and the joint analyses with line-cross model and dam-family model are presented in Table 1. For each trait, the maximum likelihood ratio test (LRT), the corresponding QTL substitution effects and most likely positions are given. Profiles of the test statistics along the chromosome for average BFT and HW are plotted in Figure 1 for line-cross analyses of male performances.

**Table 1:** QTL detections on single populations and joint QTL detections

		**Line-cross model**				**Dam-family model**		
**Trait and design**^**1**^	**N**	**LRT**^**2**^	**Pos**^**3**^**(cM)**	**CI**^**4**^**(cM)**	**Effect**^**5**^	**LRT**	**Pos (cM)**	**CI (cM)**
**(a) JXAU F2 females**								
CW (kg)	406	3.8	0	-	-	77.6	1	-
BFT at shoulder (mm)	406	3.3	62	-	-	67.7	74	-
BFT at 6–7 rib (mm)	406	12.3*	59	48-68	1.36	79.3	103	-
BFT at last rib (mm)	406	20.2***	59	51-74	1.56	81.6	59	-
BFT at hip joint (mm)	406	26.0***	61	47-69	2.35	80.6	35	-
Average BFT (mm)	406	15.6***	59	44-70	1.41	71.9	103	-
HW (kg)	406	11.3*	66	50-79	−0.08	75.8	60	-
IMF (%)	406	18.1***	59	48-67	0.13	78.4	87	-
LEA (cm^2^)	406	3.4	74	-	-	98.8	12	-
**(b) JXAU F2 males**								
CW (kg)	498	9.2*	0	0-20	−0.83	98.7	2	-
BFT at shoulder (mm)	498	18.7***	70	64-79	1.40	77.4	70	-
BFT at 6–7 rib (mm)	498	18.6***	71	65-77	1.49	61.3	70	-
BFT at last rib (mm)	498	32.6***	70	72-83	1.67	82.5	70	-
BFT at hip joint (mm)	498	63.1***	71	68-74	3.19	120.7*	71	68-76
Average BFT (mm)	498	37.5***	70	66-74	1.98	87.5	70	-
HW (kg)	498	8.1*	71	38-80	−0.06	79.3	110	-
IMF (%)	530	33.3***	74	64-78	0.24	107.2*	83	82-87
LEA (cm^2^)	473	49.0***	72	71-74	−1.38	119.5*	72	71-74
**(c) INRA F2 males**								
CW (kg)	512	7.2	46	-	-	34.6	28	-
BFT1 (mm)	516	59.9***	80	74-85	1.30	83.5***	82	78-86
BFT2 (mm)	516	73.4***	79	74-85	1.46	100.1***	84	78-86
Average BFT (mm)	516	74.1***	79	74-85	1.40	95.7***	82	75-86
HW (kg)	517	63.5***	74	72-77	−0.09	81.5***	75	73-78
IMF (%)	236	21.3***	86	77-87	0.25	40.4	83	-
LEA (cm^2^)	484	9.7*	72	47-85	−0.95	39.3	72	-
**(c) Joint F2 males**								
CW (kg)	1010	8.4*	4	0-33	−0.72	121.5	0	-
Average BFT (mm)	1010	101.8***	76	73-80	1.39	168.5***	76	-
HW (kg)	1010	66.5***	73	71-77	−0.08	136.5***	74	72-78
IMF (%)	766	41.6***	86	74-87	0.24	147.6**	83	-
LEA (cm^2^)	857	55.7***	72	71-77	−1.38	150.7**	72	-

^1^*CW* carcass weight, *BFT* backfat thickness, *HW* ham weight, *IMF* intramuscular fat content, *LEA* loin eye area. In INRA experiment, BFT1 was measured between the 3rd and the 4th lumbar vertebrae at 8 cm from the spine; BFT2 measurement was taken three vertebrae beneath the last rib at 6 cm from the mid-dorsal line.

^2^*LRT* likelihood ratio test. Significance levels: * 5% chromosome-wide significance; ** 5% genome-wide significance; *** 1% genome-wide significance.

^3^Pos = position.

^4^*CI* confidence interval of QTL position computed by a 1-LOD drop-off method.

^5^ QTL substitution effect = Chinese breed allele – European breed allele.

Based on line-cross analyses, we detected QTL for carcass weight (CW), BFT, HW, intramuscular fat content (IMF) and loin eye area (LEA) on SSCX. In the JXAU results, animals from the two sexes shared some genome-wide significant QTL for fatness traits (BFT at last rib and at hip joint, average BFT and IMF) and a suggestive QTL for HW. Despite the overlapping of confidence intervals (CIs) of the position estimates in both analyses, for males, the fatness and HW QTL were mapped at similar position (70–71 cM), distal to those mapped for females (59–66 cM). Moreover, fatness QTL seemed to exhibit stronger effects on male traits than on female traits. Effects were similar in both sexes for the HW QTL. Significant QTL effects on BFT at shoulder, LEA and CW were found only in males. The LEA QTL was located nearby the BFT QTL, whereas the CW QTL was mapped at 0 cM, far away from other QTL locations and with reduced significance level. The QTL confidence intervals (CI) were larger than 30 cM for CW, HW and IMF, and between 10 and 15 cM for the remaining traits.

From analysis of the INRA F_2_ male data, we identified highly significant QTL for BFT (79–80 cM), HW (74 cM) and IMF (86 cM) using the line-cross model. Surprisingly, no significant result for CW was obtained in the INRA male population, which is inconsistent with that obtained in the JXAU male population.

Joint analyses resulted in greater significance for tests of BFT, HW and IMF compared with individual population analyses. QTL peaks for BFT and HW were located at 76 cM and 73 cM, respectively - at an intermediate position between the QTL peaks obtained in each population. In contrast, joint analysis resulted in slightly lower significance of the CW QTL compared with the analysis of JXAU population. This may reveal either that this QTL could segregate only in the JXAU population, or that this QTL was a false positive one as the significance level was low in the JXAU analysis.

To test whether QTL effect is dependent on the population or not, we compared the models with and without population interaction for the QTL effect estimation. The interaction was not significant for any trait (*P* > 0.05). In addition, to test whether a trait was affected by 2 QTL, the LRT of 2-QTL vs. 1-QTL hypotheses were estimated using the QTLMap software (http://dga7.jouy.inra.fr/qtlmap/; [25]). There were no significant evidence for additional QTL influencing these traits (data not shown), except for IMF. Two QTL for IMF were revealed only in the INRA population (the maximum LRT_2vs1_ is equal to 57.40); they were located at 87 cM and 92 cM respectively, and exhibited opposite effects (1.95% and −0.06%, respectively) on the trait. As distinguishing the effects of linked positions requires both informative markers and informative meiosis [26], it is usually unlikely to find close significant linked QTL in linkage analyses, or the risk of pointing out statistical artifacts is high. In the present design, two genetic markers were genotyped between position 87 cM and position 92 cM, with 2/3 of the dams being heterozygous for the first one, and all for the second. However, because the number of informative meiosis is relatively limited, additional data should be accumulated to validate these two QTL.

Dam-family analyses evidenced all QTL detected in the INRA and joint population by line-cross analyses, but provided less consistent results for the JXAU population. This is probably due to that the offspring-size of each dam in the INRA population is larger (9 to 63 progeny per dam) than that in the JXAU population (2 to 22 progeny per dam), which gives greater power to familial QTL detection and thus better accuracy of QTL effects estimation in the former.

### QTL analyses in two combined dam families

We estimated the effect size of QTL for BFT in each INRA F_1_ dam family (N = 10) that had ≥30 F_2_ males. Except for the family of a F_1_ dam 910013, large and significant QTL effects were observed in families of all the other F_1_ dams including 910013’s full sisters (910002, 910009 and 910010). It is noted that these four full sisters shared the same paternal (LW) chromosome X while the 90013 inherited the other maternal (MS) chromosome. Because the segregation and effect size of SSCX QTL in F_2_ males depends on QTL genotype of their corresponding mothers (F_1_ dams), the difference in QTL effect size among the F_1_ full sisters suggests that different QTL allele occurs on MS chromosomes.

**Figure 2:**
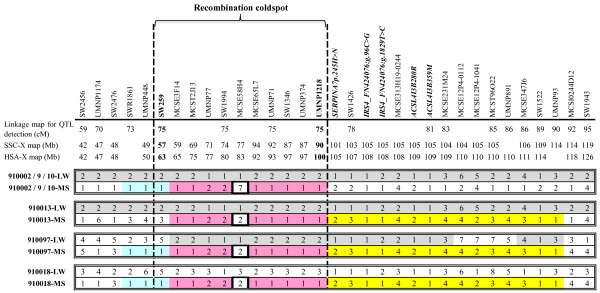
**Haplotype analysis of a subset of INRA F1 sows.** Each chromosomal segment is depicted as a series of marker alleles, ordered relatively to the joint linkage map, RH map, pig clone map and human-pig comparative map. Marker names, linkage map position (in cM), swine physical map (from Sscrofa Build 10.2 Assembly; in Mb) and human physical map (from Human Build 36 Assembly; in Mb) are indicated above the respective alleles. The region from *SW259* to *UMNP1218* (delimited by dash lines) is the recombination coldspot. Individual sows identity, as well as the breed of origin (LW = Large White; MS = Meishan), are indicated to the left of each segment. QTL were segregating in 910002 (which harbours same haplotypes as her full-sisters 910009 and 910010), but not in other three related F1 sows 910013, 910097 and 910018.

We also noticed that another two F_1_ dams (910018 and 910097) carried the same maternal haplotype as the 910013 did (Figure 2), which is identical-by-descent (IBD) over the QTL region (See Methods for details). Based on that, the above-mentioned 6 F_1_ dams were divided into two groups (group A, dams 910013, 910018 and 910097, and group B, dams 910002, 910009 and 910010) according to their maternal haplotypes. To confirm QTL segregation within MS breed, we performed QTL analyses separately for the two combined dam families A and B, which have greater statistical power of the analyses compared to individual families. Table 2 presents a striking contrast between the significances of the QTL results obtained on the two combined families. There was complete absence of QTL for BFT in the combined family A. No QTL effect on HW was detected, despite a maximum *LRT* for HW located at 72 cM, but much lower than the 5% chromosome-wise significance threshold (11.3). It should be pointed out that the contribution of each dam family to the QTL results was unequal, because of their different offspring-sizes. The three related F_1_ sows 910013, 910018 and 910097 had 30, 13 and 13 phenotyped F_2_ sons, respectively. In contrast, the combined dam family B with three F_1_ full-sisters 910002, 910009 and 910010 yielded highly-significant *LRT* for both BFT and HW at 78 and 76 cM, respectively. The joint analysis of data from families A and B including an interaction between the QTL effect and the family revealed a significant interaction between the familial origin and the QTL effect for the BFT measurements, whereas this interaction was not significant for HW (Table 2). To confirm that this difference is not due to a low power of detection in families A, 1000 simulations were performed with a QTL effect identical in the two groups of families. The interaction was significant in only 7.1% of the simulations, indicating that the significant interaction is unlikely a false positive. These results therefore suggest that F_1_ sows in the families A and B were, respectively, homozygous and heterozygous for the QTL affecting BFT and HW.

**Table 2:** QTL detections using combined dam families from INRA population

	**Combined dam family A**^**1**^			**Combined dam family B**^**2**^			**Combined dam families A and B with interaction effect**^**3**^		
**Trait**^**4**^	**N**^**5**^	**LRT**^**6**^	**Position (cM)**	**N**	**LRT**	**Position (cM)**	**LRT**	***P*value**^**7**^	**Position (cM)**
CW	56	1.1	0	129	3.0	41	-	-	-
BFT1	55	2.3	73	133	15.8**	78	18.2	0.08	80
BFT2	55	1.3	59	133	18.2**	78	19.8	0.04	80
Average BFT	55	2.2	74	133	18.4**	78	20.8	0.05	80
HW	56	5.7	72	130	25.3***	76	31.0	0.52	82

^1^The combined dam family “A” include the sub-families of three F_1_ sows: 910013, 910018 and 910097, H0 ‘no QTL effect’ *versus* H1 ‘same QTL effect in all dam families’.

^2^The combined dam family “B” include the sub-families of three F_1_ full-sisters: 910002, 910009 and 910010, H0 ‘no QTL effect’ *versus* H1 ‘same QTL effect in all dam families’.

^3^H0 ‘no QTL effect’ *versus* H1 ‘QTL effect depending on the dam family origin A or B’. Not tested for CW.

^4^For abbreviations of the traits, see Table 1.

^5^N, the number of F_2_ males in each combined family.

^6^*LRT* likelihood ratio test, ** 5% genome-wide significance; *** 1% genome-wide significance.

^7^*P* value of the test of the interaction effect.

### Haplotype analyses in the INRA F_1_ dams

The maternal (MS) and paternal (LW) haplotypes inherited by the INRA F_1_ sows within the two combined dam families A and B are shown in Figure 2 for the preliminary QTL region (between *SW2456-SW1943*). This chromosomal region encompasses a large recombination coldspot (~0 cM) that extends from *SW259* to *UMNP1218*, with a corresponding physical distance of 36.4 Mb on the human chromosome X.

As mentioned above, the MS chromosomes of the F_1_ dams from the groups A and B bear different QTL alleles. The two groups shared a very similar MS haplotype over most part of the coldspot between *MCSE3F14* and *UMNP1218* (about 34.5 Mb in length), if the mere difference of alleles at a microsatellite locus *MCSE58H4* between them is disregarded (Figure 2). Moreover, the MS haplotype consisting of three microsatellites (*SWR1861*, *UMNP448* and *SW259*) in front of the coldspot was also shared by the 910002, 910097 and 910018. In contrast, over the region *SW1426*-*UMNP93* behind the coldspot, the MS haplotype of the group A is clearly distinct from that of the group B, which matches the QTL allele distribution in the two groups. Thus, the QTL is more likely to locate downstream of the coldspot than elsewhere.

### Candidate gene analyses

*ACSL4* gene is just located downstream of the coldspot (Figure 2) and involved in lipid metabolism, so it stands out as a prime candidate gene underlying the QTL. We examined allele frequencies of the *ACSL4* SNP polymorphisms in the founder breeds and F_2_ males of the two populations (Table 3). Alleles 1 for the two SNPs were fixed in the 6 LW and 2 WD F_0_ boars, whereas two alleles for each SNP were segregating in the 6 MS and 17 ER F_0_ sows. Compared to MS, ER had higher frequency of the *ACSL4I3280R* allele 1 (0.85 vs. 0.58) but lower frequency of the *ACSL4I3B359M* allele 1 (0.32 vs. 0.58). Only three haplotypes 1–1, 1–2 and 2–1 were segregating in these Chinese sows. Haplotype 1–1 was the most frequent (>0.50) in the two F_2_ populations due to fixation in the European breeds. Haplotypes 1–2 and 2–1 showed almost equal frequencies in INRA F_2_ males, while the haplotype 2–1 was scarce in the JXAU F_2_ males, due to low frequency of allele 2 for *ACSL4I3280R* in ER.

**Table 3:** **Frequencies of SNP alleles and haplotypes in the intron3 of *ACSL4 *gene**

		***ACSL4I3B280R***		***ACSL4I3B359M***		**Haplotypes**^**1**^			
**Population**	**N**	**Allele 1 (G)**	**Allele 2 (A)**	**Allele 1 (A)**	**Allele 2 (C)**	**1-1**	**1-2**	**2-1**	**2-2**
Large White	6	1.00	0.00	1.00	0.00	1.00	0.00	0.00	0.00
White Duroc	2	1.00	0.00	1.00	0.00	1.00	0.00	0.00	0.00
Meishan	6	0.58	0.42	0.58	0.42	0.16	0.42	0.42	0.00
Erhualian	17	0.85	0.15	0.32	0.68	0.17	0.68	0.15	0.00
INRA F2 males	551	0.76	0.24	0.74	0.26	0.50	0.26	0.24	0.00
JXAU F2 males	497	0.96	0.04	0.66	0.34	0.61	0.35	0.04	0.00

^1^Haplotypes are coded as x-y, where x is the allele for the *ACSL4I3B280R* (*ACSL4* intron3:g.280G > A), and y is the allele for the *ACSL4I3B359M* (*ACSL4* intron3:g.359A > C).

Table 4 shows that *ACSL4* haplotypes exerted significant effects (*P* <0.001) on BFT, HW and IMF but not on CW. The haplotype 1–1 was associated with lower fatness and larger HW, which is contrary to the effects of haplotypes 1–2 and 2–1 on these traits. Moreover, the effects of the haplotypes 1–2 and 2–1 did not differ significantly for any trait, but differed significantly from the effects of the haplotype 1–1 (*P* < 0.05). Including SNP genotypes as fixed effect in the QTL detection analysis applied to the joint population with a line-cross model resulted in a considerable decrease in significance of the test statistic for the BFT and HW QTL, but the QTL detection remained highly significant and the QTL location did not change (Table 5). However, when the *ACSL4* SNP haplotypes were included as fixed effects in a similar analysis, all QTL became non-significant. Even so, the F_1_ sows in the combined families A and B were found to have the same *ACSL4* haplotype pattern (Figure 2).

**Table 4:** ***ACSL4 *haplotype effects on phenotypes in the joint population**

**Traits**^**1**^	***P*value**^**2**^	**Haplotype**^**3**^	**Least-square means**^**4**^	**SE**
CW (kg)	0.57	1-1	0.05	0.31
		1-2	−0.24	0.42
		2-1	0.31	0.62
Average BFT (mm)	<0.001	1-1	−1.02^a^	0.22
		1-2	1.19^b^	0.30
		2-1	1.38^b^	0.44
HW (kg)	<0.001	1-1	0.05^a^	0.01
		1-2	−0.06^b^	0.02
		2-1	−0.07^b^	0.03
IMF (%)	<0.001	1-1	−0.11^a^	0.04
		1-2	0.15^b^	0.05
		2-1	0.15^b^	0.09

^1^ For abbreviations of the traits, see Table 1.

^2^*P*-values for the test of a linear model accounting for fixed effects of the haplotype, sire and dam.

^3^Haplotypes are coded as x-y, where x is the allele for the SNP *ACSL4I3B280R*, and y is the allele for the SNP *ACSL4I3B359M*.

^4^Values within a trait with different superscripts differed significantly (*P* < 0.05). A value marked as ^a^ is significantly different from those marked as ^b^.

**Table 5:** **Joint QTL detections for phenotypes corrected for the *ACSL4 *SNPs genotypes and haplotypes**

	***ACSL4I3B280R***		***ACSL4I3B359M***		**Haplotype**	
**Traits**^**1**^	**LRT**^**2**^	**Position (cM)**	**LRT**	**Position (cM)**	**LRT**	**Position (cM)**
CW (kg)	11.4	7	10.5	0	10.8	2
Average BFT (mm)	43.7*	76	28.6*	75	6.2	110
HW (kg)	30.9*	73	28.8*	73	7.7	72

^1^For abbreviations of the traits, see Table 1.

^2^*LRT* likelihood ratio test. * 5% chromosome-wide significance.

The segregation of several SNPs in the other two positional candidate genes *IRS4* and *SERPIN7* were also analyzed in the INRA F_0_ and F_1_ animals. Except 2 MS F_0_ sows that are heterozygous (CG) for a SNP FN424076:g.96C > G in the *IRS4* gene, other 4 MS F_0_ sows and 6 LW F_0_ boars are all homozygotes (CC). For another SNP FN424076:g.1829T > C in this gene, all MS F_0_ sows were homozygous for the “C” allele while LW F_0_ boars were homozygous for the “T” allele. Similarly, alternative alleles for three SNPs p.245H > N (or c.733A > C, AY550250:g1838A > C), AY550250:g2276A > G and AY550250:g2324A > G in the *SERPIN7* gene were fixed between the MS (CC, AA and AA) and LW (AA, GG, GG) founders, thus leading to alternative haplotype (C-A-A and A-G-G) were fixed between them. Moreover, the F_1_ sows in combined families A and B shared the same genotype of the test SNPs in the two genes (Figure 2).

## Discussion

The first purpose of this study is to fine map the SSCX QTL for fatness and muscling traits. From the primary scan in the INRA population, the CI of the QTL for BFT and HW were approximately 15 cM wide [4]. In this study, they were dramatically reduced to merely 7 cM (Table 1) through joint analysis of both INRA and JXAU populations with new information of high-density markers commonly genotyped for them. However, the current QTL intervals still contain a large recombination coldspot which spans from *SW259* to *UMNP1218* (~36.4 Mb vs. 0 cM), therefore further refinement of these QTL by linkage analysis seems impossible.

Fortunately, the distribution of the chromosomes in the INRA population permitted to study two groups of related F1 dams. Despite unbalanced number of progeny in the two groups, we conclude that they have contrasted genotypes for the QTL. Indeed, the power estimation for the detection of the interaction between the QTL effect and the groups indicates that it is highly unlikely that the QTL is not detected in “homozygous dams” families due to a lack of power in this group. From this conclusion, the haplotype analysis of these two groups with different QTL genotypes enabled us to estimate the most likely position of the QTL. The full sisters 910002 and 910013 carried the same LW chromosome X but different MS X chromosomes bearing different QTL alleles. Because 910013 is homozygous for the SSCX QTL, its MS and LW chromosomes X share a similar QTL allele “q” associated with low BFT and high HW traits. In contrast, the heterozygote 910002’s MS chromosome X contains another QTL allele “Q”. In the coldspot region between *MCSE3F14* and *UMNP1218* (about 34.5 Mb in length), 910013 carries a haplotype defined as Hap1, associated thus to a “q” allele, whereas 910002 carries a Hap2 haplotype associated with a “Q” allele. Noticeably, Hap1 and Hap2 MS haplotypes over most part of the coldspot are quite similar except for alleles at a microsatellite locus *MCSE58H4* (Figure 2). Interestingly, 20 out of 23 F1 sows of the French families inherited the MS Hap1 (“q” allele) and the BFT QTL explained about 40% of phenotypic variance in the whole INRA F_2_ male population [4]. Even if the limited numbers of their offspring of most F1 sows do not allow individually determining their QTL genotype, a large part of F1 sows are thus supposed to be heterozygous for the QTL, i.e. having a “Q” MS allele, despite harboring a Hap1 haplotype. Globally, this region of coldspot of recombination is thus extremely poorly polymorphic, and the two closely related haplotypes do not co-segregate with the QTL haplotypes. It is thus very likely that the causal mutation affecting BFT traits is located outside of the region *MCSE3F14*-*UMNP1218* corresponding to the coldspot of recombination.

In addition, another IBS haplotype spanning *SWR1861*-*SW259* interval ahead of the coldspot was also found on the MS chromosomes X of the segregating (910002) and non-segregating (910097 and 910018) sows. If the QTL was located in this interval, 910097 and 910018 sows would share the same “Q” allele on their MS chromosomes as the 910002. As 910097 and 910018 are homozygous at the QTL, their LW chromosomes should then harbor the “Q” allele, which is not likely. Thus, we can conclude from the haplotype analysis that it is not so likely to have the QTL just at the upper boundary of the coldspot (in blue on Figure 2). However, we cannot exclude that the QTL can be farer on the upper left area, where no common haplotypes with the used microsatellites was seen between individuals from the groups A and B.

In the region *SW1426-UMNP93*, two different MS haplotypes were observed between the full sisters 910002 and 910013 who had different MS QTL alleles, as well as between their belonged groups (A and B). More importantly, we previously evidenced that the three related F_1_ dams (910013, 910097 and 910018) inherited the same MS haplotype over the region from their recent ancestor; that is, their MS haplotypes are identical by descent (IBD) rather than mere IBS to each other [24]. Similarly, the MS haplotypes carried by the full sisters 910002, 91009 and 910010 are also IBD. Because of the perfect match between allelic and IBD-haplotypic distribution among these MS chromosomes, we believe that the region downstream the coldspot (in yellow on Figure 2) is most likely to contain the QTL, which is also in agreement with the likely location of the QTL detected in the INRA population (Figure 1).

Following the study of Pérez-Enciso et al. [14], we managed to find some clues for supporting either the hypothesis of single pleiotropic QTL or the hypothesis of multiple linked QTL for the investigated traits. However, in our study the only linked QTL test significant was for IMF, suggesting that two positions separated by only 5 cM have joint and opposite effects on the trait. Despite the presence of two highly informative markers between these positions, the power to discriminate between these two positions is not high in our study due to limited number of recombination events. This result needs confirmation to validate that it is not an artifact [26]. Čepica *et al.*[2] reported a genome-wide significant QTL for CW that co-localized with QTL for BFT at the centromeric region of SSCX in a Wild Boar × MS cross. However, QTL for CW detected in the JXAU F_2_ males only reached a suggestive level and was mapped at 0 cM, far away from QTL for other traits. Moreover, there was an absence of QTL for CW in the INRA F_2_ males. These results indicate that the present QTL for fatness or HW has probably negligible effect on CW.

Pérez-Enciso *et al.*[14] reported that at least two distinct regions segregate on SSCX in different populations, one in the neighborhood of *SW259/SW1994* markers, with an effect on ham weight and carcass length, and another one between markers *SW2476* and *SW1943*, with primary effects on fatness and shoulder weight. It must be noted that the marker order *SW259/SW1994* (74.4 cM) - *SW2476* (77.6 cM) - *SW1943* (87.4 cM) on the USDA-MARC porcine genetic map was inconsistent with the order *SW2476* - *SW259*-*SW1994* - *SW1943* on RH map and pig clone map [24,27]. The present result of QTL analyses in the INRA F_2_ males showed that the HW QTL peak was located only 5 cM upstream of the BFT QTL peak (Table 1), which is in agreement with Pérez-Enciso *et al.*[14] and Čepica *et al.*[2]. Even so, we couldn’t discriminate the HW QTL from the BFT QTL because their CI overlapped. Indeed, as shown in Figure 1, the peak in the test statistics curve for the BFT QTL was much broader than that for the HW QTL detected in the INRA F_2_ males, and the latter was within the former. Moreover, the QTL for HW and BFT found in the JXAU F_2_ males were located at the same position (71 cM), very close to the location (74 cM) of the HW QTL detected in the INRA F_2_ males. Thus, although CI overlapped and tests for 2 linked QTL were not significant, our results, on one hand, are in agreement with the previous suggestions that two QTL exist: one proximal to *UMNP1174* and another proximal to *SW1426* (Figure 1), and on the other hand, they indicate the former QTL influence both BFT and HW rather than only HW.

It is interesting to compare the sizes of QTL effects on the same trait between the two populations. We found that the SSCX QTL for average BFT could explain 5.7% of phenotypic variation in the JXAU F_2_ population, which is lower than 35.9% and 6.2% of those explained by QTL mapped on SSC7 and SSC4, respectively [28]. In contrast, the SSCX QTL detected in the INRA population showed markedly stronger effect on BFT than the SSC4 and SSC7 QTL [4]. Nevertheless, high significance and similar map location of the BFT QTL on SSCX were found in the two populations, suggesting the existence of common QTL between them. As to the QTL for HW and LEA on SSCX, their significance levels differed largely between populations (Table 1). Given the same QTL shared by the two populations, these discrepancies were probably due to the differences between them in epistatic QTL [29], QTL allele segregation pattern of the founder breeds, environment effect and/or trait measurements. Despite these discrepancies, we detected no interaction between QTL effects and population, and the estimates of substitution allele effects in the two populations were close (Table 1).

The results of the QTL analysis and haplotype analysis in two combined F_1_ dam families indicate QTL segregation within the MS breed rather than the LW breed, which is expected since the LW instead of the MS has been selected for lean growth over decades as a commercial line. The Chinese MS pig with excess body fat is often used in breeding programs in order to take advantage of its prolificacy, while during the process, how to avoid the disadvantages of its excessive fatness and low growth rate have to be considered. The segregation of SSCX QTL for BFT and HW within Meishan breed provides an opportunity for us to make effective use of Meishan chromosome X in crossbreeding and to increase the frequency of the favorable alleles in the purebred by marker assisted selection.

*ACSL4* is located at 80.5 cM, in close proximity to the most likely position of QTL for BFT identified in the INRA population. This gene showed consistent and multiple significant associations at the single SNP (data not shown) and haplotype levels in the two populations (Table 4). However it is obvious that the two *ACSL4* SNPs are not the causal mutation(s) because the segregating and non-segregating F_1_ sows had the same SNP genotype. But their haplotypes should be linked with the causal mutation(s) because the QTL disappear when accounting for the gene haplotypes. Therefore, we cannot preclude that a polymorphism in *ACSL4* mRNA sequence or its cis-acting elements may result in the QTL effects. Mercadé *et al.*[15] sequenced most of the region of the *ACSL4* mRNA in multiple breeds, and identified 10 polymorphisms within the 3’-UTR region, all of which formed only two haplotypes. Further, they found that the haplotype 1 (DQ144454:g.2274A-2645G-2782G-2933delete-2934delete-3272-C-3590G-3591T-3862T-4074A) fixed in the MS breed was at high frequency (0.95) in the LW breed. As the MS and LW pigs were used as founders in the INRA population, these polymorphisms or haplotypes could not be responsible for our observed effects on BFT.

The candidate genes *IRS4* and *SERPINA7* are also within our refined QTL region. Previous studies [16-18] reported significant associations between the *IRS4* SNPs (FN424076:g.96C > G and FN424076:g.1829T > C) and BFT, as well as between a missense mutation p.245N > H in the *SERPINA7* gene and BFT. In this study, the SNP FN424076:g.96C > G can be firstly excluded as a causal mutation, because its “C” allele was the major allele in both the MS and LW founders that likely carry different QTL alleles. Furthermore, the other SNPs, like the two SNPs in *ACSL4*, were not co-segregating with QTL alleles between the combined families A and B, so they are unlikely to be causal mutation either.

Despite the lack of supporting evidence for the polymorphisms in the three candidate genes underlying the target QTL, further research is needed to identify their potential variations in DNA sequence (e.g. copy number variation), DNA methylation and gene expression levels.

## Conclusions

This study displays narrower CI for all investigated QTL and suggests that these QTL are likely to locate outside of the large recombination coldspot from marker *MCSE3F14* to *UMNP1218*, leading to great reduction of the number of candidate genes. Moreover, this study is also consistent with the previously reported existence of at least two adjacent QTL regions, one proximal to *UMNP1174*, with pleiotropic effects on fatness and muscling traits, and another one proximal to *SW1426* that seems to mainly influence fatness. Some *ACSL4* polymorphisms are significantly associated with BFT and HW phenotypes in the two populations, suggesting that the gene may be involved in the studied QTL effects, or at least linked to the causal variant. Notably, QTL for BFT and HW segregate within the MS breed. These findings may contribute to the identification of causal genes underlying these QTL and the effective use of Meishan pigs with favorable QTL alleles in crossbreeding programs.

## Methods

### Animals and traits

Data from the INRA and JXAU population were used. Details about raising and management of the two populations and the traits recorded were previously described elsewhere [1,4,9,28]. Briefly, the INRA population was created by the cross between 6 Large White boars and 6 Meishan sows. The 488 F_2_ castrated males generated from 6 F_1_ boars and 23 F_1_ sows were slaughtered at approximately 180 days of age and submitted to a standardized cutting of the carcass. The JXAU population was established by crossing 2 White Duroc boars to 17 Erhualian sows, from which 9 F_1_ boars and 59 F_1_ sows were used to produce 1912 F_2_ progeny. At 240 ± 3 days of age, 548 F_2_ castrated males and 481 F_2_ females were slaughtered and phenotyped for carcass composition traits.

In the INRA experiment, carcass weight (CW), two backfat thicknesses (BFT1 and BFT2), ham weight (HW) and loin eye area (LEA) were obtained after slaughter and analyzed in the current study. BFT1 was measured between the 3rd and the 4th lumbar vertebrae at 8 cm from the spine. BFT2 and LEA measurements were taken simultaneously beneath the last rib at 6 cm from the mid-dorsal line. Of all slaughtered F_2_ males, only 236 individuals, offspring from 4 F_1_ boars and 16 F_1_ sows were measured for intramuscular fat content (IMF). In the JXAU experiment, the traits analyzed were CW, BFTs at shoulder, 6–7 ribs, last rib and hip joint, HW, IMF and LEA. An average BFT was computed from BFT at last rib and 6–7 ribs in the JXAU experiment, and from BFT1 and BFT2 in the INRA experiment, these two locations being the closest in the two experiments.

The average BFT, CW, HW and IMF were further used for joint analyses of the two populations. These traits were first validated as 1) being recorded similarly in the two populations, 2) showing similar coefficients of variation in the two populations. However, due to differences in age at slaughter and measurements, differences in means and standard deviations were observed, so the traits were centered to zero and standardized within experiments prior to the joint analyses.

All animal experiments were conducted in accordance with European Communities Council Directive of 24 November 1986 (86/609/EEC) and the Guidelines for the Care and Use of Animal established by the Ministry of Science and Technology of P.R. China (1988).

### Marker genotyping and linkage map construction

Primer sequences for 13 newly developed markers (Additional file 1: Table S1) were designed using Primer3 software (http://primer3.sourceforge.net/). To confirm the locations of all used markers, they were mapped onto the INRA-University of Minnesota porcine radiation hybrid (IMpRH) panel using IMpRH sever [30,31]. PCR were typically performed in a 10 μl reaction volume containing 20–25 ng of template DNA, 1 × PCR buffer, 200 uM each dNTP, 0.25 uM each of forward and reverse primer (forward primer were labeled with fluorescent tags), and 0.25 U Taq polymerase (AmpliTaq Gold DNA polymerase; Applied Biosystems). For some forward primers without fluorescent tags, an M13 adaptor (5’-GTTTTCCCAGTCACGACGTTG-3’) was added to their 5’ ends, as described by Schuelke [32]. In this case, the concentrations of forward, reverse and M13 primers in PCR reaction were adjusted to 0.1, 0.15 and 0.15 uM, respectively. The typical PCR profiles included an initial denaturation at 94°C for 5 min followed by 35–45 cycles of 94°C for 30 sec, annealing temperatures (50–60°C) for 30 sec and 72°C for 30 sec, with a final extension at 72°C for 20 min. PCR products of microsatellites were analyzed for fragment length using ABI3130 or ABI3730 sequencers and GeneMapper 3.7 software (ABI, Foster City, USA). The *ACSL4I3B280R* and *ACSL4I3B359M* were genotyped by PCR-RFLP, after digestion by enzyme *MspI* and *Tsp509I*, respectively. Length polymorphism of *SLC25A5I2B102DE* was assessed by agarose gel (3%) electrophoresis.

Female-specific linkage map was constructed using CRIMAP 2.4 [33], with the 22 markers’ genotypes in both populations. Resulting recombination fractions were then converted into map distances using the Haldane’s mapping function. The joint linkage map obtained on the two populations covers 110 cM, with an average intermarker distance of 2.1 cM within the QTL region *SW2456*-*SW1943*. Markers *SW259*, *SW1994*, *SW1426* and *SW1522* were in different position/order compared with the USDA-MARC linkage map, but their order was retrieved using the IMpRH (7000-rad) panel and the reference map INRA2006 (http://rhdev.toulouse.inra.fr/Do=Maps). Details of the markers and linkage map are given in Additional file 1: Table S1 and Figures 1 and 2), respectively. Because four microsatellites *SW259*, *SW1994*, *SW1943*, *UMNP1218* were mapped at the same position (74.9 cM), only the most informative marker in terms of heterozygosity in the F1 dams, *SW259*, was used in the QTL analyses. *ACSL4* was located at 80.4 cM and only one SNP (*ACSL4I3B359M*) in it, the more informative one, was included in the QTL analysis.

### Statistical analyses

#### QTL analyses

QTL detection was performed using the QTLMap software (http://dga7.jouy.inra.fr/qtlmap/; [25]). A line-cross model and a dam-family model were both applied. QTL positions were computed on the joint linkage map. For the line-cross model, the general univariate model for all traits was:

$$Y_{\mathrm{ijk}}=\mu_{\mathrm{ij}}+\mathrm{effec}t_{l}+P_{\mathrm{jk}}S+e_{\mathrm{ijk}}$$

where *y*_*ijk*_ is the record for individual *k* from sire *i* and dam *j*; *μ*_*ij*_ is the sire *i* and dam *j* family mean; *effect*_*l*_ is a set of fixed effects and covariables estimated for the F_2_ population *l* (*l* = INRA or JXAU); *S* is the substitution effect for the QTL alleles; the coefficient *P*_*jk*_ is the probability of the *k*th individual inheriting the allele of Chinese or Western breed origin from the dam *j*; *e*_*ijk*_ is the residual value of mean zero and standard deviation *σ*_*i*_. *effect*_*l*_ covered batch as fixed effect and carcass weight as a covariant for BFT and HW. In the dam-family analyses, a specific substitution effect *S*_*k*_ was estimated for each dam family.

The populations were initially analyzed separately. For the JXAU population, the QTL analyses were performed separately for each sex (F_2_ males and females). F_2_ males and females were analyzed separately because they have different number of X chromosomes, meaning potentially different expression of QTL effect, and the random inactivation of one chromosome X in females may cause potential bias of the estimated QTL effects and location. For joint analyses, only F_2_ males were available in both experiments. We grouped Chinese MS and ER as one fixed “breed” and the Western breeds LW and WD as another. The single-QTL model with population interaction was also tested to investigate whether the QTL effects were significantly different in the populations. The maximum likelihood for the interaction model was compared to the maximum likelihood for the model with no interaction [12] to test whether the latter could be rejected.

This study focuses on a single chromosome where QTL have been detected in the two populations; thus it might be argued that a genome-wide significance threshold is too stringent. However, we expect that the future joint analysis may be used to scan the entire genome and one of the major aims of this study is a comparison with genome scans based on individual studies. Thus, for the sake of comparison, we used a genome-wide significance threshold. The genome-wide thresholds were derived from chromosome-wide significance levels, using an approximate Bonferroni correction: *P*_*Genome*-*wide*_ = 1 - (1 - *P*_*Chromosome*-*wide*_)^1/*r*^, in which *r* was the number of chromosomes in the pig genome [34]. Chromosome-wide thresholds for each trait were estimated from 2000 simulations under the null hypothesis. Following Lander and Botstein [35], approximate confidence intervals (CI) were set for QTL locations using the one-LOD drop-off method.

#### Haplotype analyses of F_1_ sows

The phase of the paternally and maternally inherited chromosomes for F_1_ sows were constructed using the Gemma software (https://www-lgc.toulouse.inra.fr/internet/index.php/Tools/Gemma.html). We previously reported that there was a significant heterogeneity in the recombination rate among the F_1_ sows in the region *SW1426*-*SW1943* within QTL interval, and inferred that this heterogeneity was associated with maternal haplotypes of Chinese origin [24]. In the INRA population, three F_1_ full-sisters (910002, 910009 and 910010) inheriting the same maternal (MS) haplotype showed significantly higher recombination rates in the region, compared with their other full-sister 910013 who carried an alternative maternal haplotype. Meanwhile, two related F1 sows 910018 and 910097 shared the same maternal haplotype over most of the QTL region with the 910013. This haplotype was proved to be identical by descent (IBD) inherited from one of their recent common ancestors [24]. Hence, we divided the 6 F_1_ dam families into two groups (combined family “A” for the families sharing the 910013’s haplotype and combined family “B” for the three other full sisters) according to their maternal haplotypes. QTL effects were estimated again within each group. When the 1-QTL model was significant, a further analysis to test the existence of 2- linked QTL was applied.

#### Candidate gene analyses

*ACSL4* haplotype effect on the traits was calculated by regressing phenotypes on haplotype using a variance analysis (SAS Inst. Inc., Cary, NC) including fixed effects of haplotype, sire and dam on phenotypes pre-corrected for previously mentioned fixed effects. In addition, we performed QTL analyses under a combined QTL linkage model [15], by running QTL detection in a model with fixed effects of the *ACSL4* SNP genotype or haplotype effects.

## Competing interests

The authors declare that they have no competing interests.

## Authors’ contributions

JM developed markers, performed the genotyping, and drafted the manuscript. HG and JB performed the statistical analyses and HG helped edit the manuscript. NI reviewed all the genotypes and checked marker Mendelian inheritance. JM, YD, BG and WH carried out the phenotyping and genotyping of the JXAU population. HM determined the SNP genotypes of the INRA F0 and F1 animals for the candidate genes. JR assisted in the study design. LH and DM conceived the study and co-supervised the work. All the authors have read and approved the final manuscript.

## Supplementary Material

Additional file 1: Table S1 Mapping information about 46 markers including 38 microsatellites and 8 SNPs in four genes (additional gene-based and BAC-based SNPs are omitted here). Click here for file
