# Supplementary material for: Fine mapping of fatness QTL on porcine chromosome X and analyses of three positional candidate genes
Source: BMC Genet. 2013 Jun 1;14:46. doi: 10.1186/1471-2156-14-46 (PMC3691627; doi:10.1186/1471-2156-14-46)
Supplement: Additional file 1: Table S1 — Mapping information about 46 markers including 38 microsatellites and 8 SNPs in four genes (additional gene-based and BAC-based SNPs are omitted here). [file 1471-2156-14-46-S1.doc]

**Table S1**

| Marker names^1^ | RH map^2^ | SSC-X physical map (Mb)^3^ | HSA-X physical map (Mb)^4^ | Current genetic map (Haldane; cM) | USDA -MARC genetic map (cM)^5^ | References^6^ |
| --- | --- | --- | --- | --- | --- | --- |
| SW949 | 2126 | 144.12& 144.25 |  |  | 0 | USDA-MARC map |
| **SW980** | 1861 | 9.07 | 11.38 | 0 | 11.9 | USDA-MARC map |
| **SW1903** | 1588 | 23.06 | 25.47 | 34.7 | 33.0 | USDA-MARC map |
| **SW2456** | 1345 | 42.24 | 42.14 | 58.6 | 55.4 | USDA-MARC map |
| **UMNP1174** | 1253 | 47.41 | 47.22 | 69.7 |  | Fahrenkrug *et al.* 2005 |
| SW2476 | 1250 | 47.55 | 48.32 |  | **77.6** | USDA-MARC map |
| **SWR1861** | 1211 |  |  | 72.8 | 65.7 | USDA-MARC map |
| UMNP448 | 1200 | 49.20 | 50.42 |  |  | Krause *et al.* 2002 |
| **SW259** | 1045 | 57.28 | 63.35 | 74.9 | **74.4** | USDA-MARC map |
| MCSE3F14 | 1093 | 58.89 | 65.3 |  |  | Ma *et al.* 2010 |
| MCST2J13 |  | 69.11 | 74.74 |  |  | Ma *et al.* 2010 |
| UMNP77 |  | 70.97 | 77.04 |  |  | Fahrenkrug *et al.* 2005 |
| **SW1994** | 1007 | 73.84 | 79.92 | 74.9 | **74.4** | USDA-MARC map |
| MCSE58H4 | 983 | 77.43 | 83.46 |  |  | Ma *et al.* 2010 |
| MCSE65L7 | 932 | 93.76 | 91.66 |  |  | Ma *et al.* 2010 |
| **UMNP71** | 905 | 92.30 | 93.18 | 74.9 |  | Korwin-Kossakowska *et al.* 2002 |
| SW1346 |  | 86.76 | 96.70 |  | **74.4** | USDA-MARC map |
| UMNP374 | 840 | 86.86 | 97.00 |  |  | Krause *et al.* 2002 |
| **UMNP1218** | 800 | 90.18 | 99.84 | 74.9 |  | Fahrenkrug *et al.* 2005 |
| *SERPINA7p.245H>N* |  | 101.07 | 105.16 |  |  | Nonneman *et al.* 2005 |
| *SERPINA7_AY550250:g2276A>G* |  |  |  |  |  | Nonneman *et al.* 2005 |
| *SERPINA7_AY550250:g2324A>G* |  |  |  |  |  | Nonneman *et al.* 2005 |
| **SW1426** | 710 | 102.85 | 107.08 | 77.9 | **71.7** | USDA-MARC map |
| MCSE313H19.0244 |  | 105.11 | 108.38 |  |  | Ma *et al.* 2010 |
| *IRS4_FN424076:g.96C>G* |  | 104.77 | 107.87 |  |  | Masopust *et al.* 2011 |
| *IRS4_FN424076:g.1829T>C* |  | *104.77* | *107.87* |  |  | *Masopust et al. 2011* |
| ***ACSL4I3B280R*** |  | 105.40 | 108.58 | 80.8 |  | Ma *et al.* 2010 |
| ***ACSL4I3B359M*** | 687 | 105.40 | 108.58 | 80.8 |  | Ma *et al.* 2010 |
| **MCSE231M24** | 682 | 103.94 | 109.41 | 82.0 |  | Ma *et al.* 2010 |
| MCSE12P4.0112 |  | 104.39 | 109.65 |  |  | Ma *et al.* 2010 |
| MCSE12P4.1041 |  | 104.46 | 109.80 |  |  | Ma *et al.* 2010 |
| **MCST96O22** | 658 | 104.61 | 110.22 | 85.1 |  | Ma *et al.* 2010 |
| **UMNP891** | 656 |  | 110.23 | 85.1 |  | Fahrenkrug *et al.* 2005 |
| **MCSE347J6** | 646 | 105.98 | 110.63 | 86.1 |  | Ma *et al.* 2010 |
| **SW1522** | 595 | 108.69 | 113.73 | 89.2 | **55.4** | USDA-MARC map |
| **UMNP93** | 540 | 113.94 |  | 90.2 |  | Korwin-Kossakowska *et al.* 2002 |
| UMNP870 | 522 | 114.32 | 117.67 |  |  | Fahrenkrug *et al.* 2005 |
| **MCSI0244D12** | 506 | 113.92 | 118.15 | 92.3 |  | Ma *et al.* 2010 |
| ***SLC25A5I2B103DE*** |  | 113.74 | 118.36 | 93.3 |  | Čepica *et al.* 2001 |
| UMNP1008 | 475 | 113.33 | 118.75 |  |  | Fahrenkrug *et al.* 2005 |
| **SW1943** | 440 | 119.45 | 126.14 | 95.3 | 87.4 | USDA-MARC map |
| **SW1608** | 304 | 125.26 | 132.31 | 109.0 | 101.9 | USDA-MARC map |
| SW707 | 286 | 126.20 |  |  | 107.9 | USDA-MARC map |
| SW2137 | 273 | 126.81 |  |  | 108.1 | USDA-MARC map |
| S0218 | 211 | 130.54 |  |  | 114.4 | USDA-MARC map |
| SW2588 |  | 140.34 | 150.01 |  | 128.4 | USDA-MARC map |

^1^Twenty-two markers in bold were genotyped in both INRA and JXAU F2 population and included in the current genetic map. SNPs in the *SERPINA7*, *IRS4*, *ACSL4* and *SLC25A5* genes are shown in italic. The SNP *SERPINA7p.245H>N* (underline) is a missense mutation, causing 245th amino acid change in SERPINA7, whereas it was mistakenly denoted as His226Asn in the original paper of Nonneman *et al.* (2005). The most likely order of all markers was determined by combination of common order shared by RH, physical and genetic maps.

^2^Positions of markers were determined by using IMpRH (7000-rad) panel and the reference map of INRA2006 (<http://rhdev.toulouse.inra.fr/Do=Maps>).

^3^Marker locations on the Sus Scrofa Build 10.2 Assembly.

^4^The full sequences of markers were submitted to “Blast all submitted clone” analysis (http://www.sanger.ac.uk/cgi-bin/blast/submitblast/s_scrofa) for identification of clones used in pig genome assemblies. Hit locations of these clones on Human Assembly Build 36 have been presented on the Sanger web (<http://www.sanger.ac.uk/cgi-bin/Projects/S_scrofa/WebFPCreport.cgi>).

^5^Bold numbers indicate that the markers (*SW2476*, *SW259*, *SW1994*, *SW1426* and *SW1522*) were in reverse order on USDA-MARC map vs. the current genetic maps or other maps.

^6^The references cited here are listed below:

1. Fahrenkrug S, Wagner M, Morrison L, Alexander LJ: **Map assignments of 373 previously unreported porcine microsatellites**. *Anim Genet* 2005, **36**(1):76-86.
2. Krause E, Morrison L, Reed KM, Alexander LJ: **Radiation hybrid mapping of 273 previously unreported porcine microsatellites**. *Anim Genet* 2002, **33**(6):477-485.
3. Ma J, Iannuccelli N, Duan Y, Huang W, Guo B, Riquet J, Huang L, Milan D: **Recombinational landscape of porcine X chromosome and individual variation in female meiotic recombination associated with haplotypes of Chinese pigs**. *BMC Genomics* 2010, **11**:159.
4. Korwin-Kossakowska A, Reed KM, Pelak C, Krause E, Morrison L, Alexander LJ: **Radiation hybrid mapping of 118 new porcine microsatellites**. *Anim Genet* 2002, **33**(3):224-227.
5. Nonneman D, Rohrer GA, Wise TH, Lunstra DD, Ford JJ: **A variant of porcine thyroxine-binding globulin has reduced affinity for thyroxine and is associated with testis size**. *Biol Reprod* 2005, **72**(1):214-220.
6. Masopust M, Vykoukalova Z, Knoll A, Bartenschlager H, Mileham A, Deeb N, Rohrer GA, Cepica S: **Porcine insulin receptor substrate 4 (IRS4) gene: cloning, polymorphism and association study**. *Mol Biol Rep* 2011, **38**(4):2611-2617.
7. Cepica S, Rohrer GA, Knoll A, Masopust M, Malek O: **Linkage mapping of four genes (OTC, SERPINA7, SLC25A5 and FMR1) on porcine chromosome X**. *Anim Genet* 2001, **32**(2):106-109.
